# The PstI/RsaI and DraI polymorphisms of *CYP2E1 *and head and neck cancer risk: a meta-analysis based on 21 case-control studies

**DOI:** 10.1186/1471-2407-10-575

**Published:** 2010-10-22

**Authors:** Kefu Tang, Yang Li, Zhao Zhang, Yunmin Gu, Yuyu Xiong, Guoyin Feng, Lin He, Shengying Qin

**Affiliations:** 1 Bio-X Center, Key Laboratory for the Genetics of Developmental and Neuropsychiatric Disorders (Ministry of Education), Shanghai Jiao Tong University, Shanghai, China; 2 Institute for Nutritional Sciences, Shanghai Institutes of Biological Sciences, Chinese Academy of Sciences, Shanghai, China; 3 Institutes of Biomedical Sciences, Fudan University, Shanghai, China; 4 Shanghai Institute of Mental Health, Shanghai, China

## Abstract

**Background:**

*CYP2E1 *encodes a member of the cytochrome P450 superfamily of enzymes which play a central role in activating and detoxifying many carcinogens and endogenous compounds thought to be involved in the development of cancer. The PstI/RsaI and DraI polymorphism are two of the most commonly studied polymorphisms of the gene for their association with risk of head and neck cancer, but the results are conflicting.

**Methods:**

We performed a meta-analysis using 21 eligible case-control studies with a total of 4,951 patients and 6,071 controls to summarize the data on the association between the *CYP2E1 *PstI/RsaI and DraI polymorphism and head and neck cancer risk, especially by interacting with smoking or alcohol.

**Results:**

Compared with the wild genotype, the OR was 1.96 (95% CI: 1.33-2.90) for PstI/RsaI and 1.56 (95% CI: 1.06-2.27) for DraI polymorphism respectively. When stratified according to ethnicity, the OR increased in the Asians for both polymorphisms (OR = 2.04, 95% CI: 1.32-3.15 for PstI/RsaI; OR = 2.04, 95% CI: 1.27-3.29 for DraI), suggesting that the risk is more pronounced in Asians.

**Conclusion:**

Our meta-analysis suggests that individuals with the homozygote genotypes of PstI/RsaI or DraI polymorphism might be associated with an increased risk of head and neck cancer, especially in Asians.

## Background

Squamous cell carcinoma of head and neck (HNSCC), including the oral cavity, pharynx, and larynx, is the sixth most common cancer in the world [1]. Epidemiological studies have shown that this type of cancer is one of those most strongly related to environmental factors, such as tobacco smoking and alcohol consumption. Many chemical carcinogens present in tobacco and alcohol undergo metabolic activation by phase I enzymes, in particular the cytochrome P450 (CYP). The activated metabolites are subsequently subjected to other detoxification steps by phase II enzymes such as glutathione S-transferases (GSTs) and N-acetyltransferases (NATs). It is hypothesized that part of the susceptibility to head and neck cancer may be determined by the inter-individual differences in the bioactivation of procarcinogens and detoxification of carcinogens. *CYP2E1*, which is a major component of the microsomal system involved in the metabolism of ethanol and acetone among phase I enzymes, has been widely studied as a cause of susceptibility to head and neck cancer [2,3].

The *CYP2E1 *gene, located on chromosome 10q26.3, is constitutively expressed in human liver and is responsible for the catalysis of xenobiotic. The protein encoded by this gene specifically activates N-nitrosamines and benzene, contained in cigarette smoke [4], and catalyses molecular oxygen to active oxygen forms (e.g., superoxide anion radical, hydrogen peroxide, etc.), which lead to intensified lipid and protein peroxidation, DNA damage, and ultimately carcinogenesis [5]. Several functional polymorphisms have been reported for the *CYP2E1 *gene. Two point mutations in the 5'-flanking region (PstI, RsaI), which are in complete linkage disequilibrium, was found to be associated with higher-transcription and increased enzyme activity [6]. These mutations generate the *CYP2E1 *wild (c1) allele and the less common (c2) allele and have been reported as conferring higher risk for developing oral, pharyngeal [7] and lung cancer [8]. Another important polymorphism detectable with DraI in intron 6 is T7632A (rs6413432), a mutation of T to A, which is reported to enhance transcription of the *CYP2E1 *gene [9]. Polymorphisms in *CYP2E1 *are therefore believed to be risk factors for HNSCC.

A number of studies have investigated the associations between *CYP2E1 *polymorphisms and HNSCC susceptibility. However, these studies have yielded contradictory results, with some studies showing a significant association, while others showing no such association. Such inconsistency could be due to the small effect of the polymorphism on HNSCC risk and the relatively small sample-size in each of the published studies. We therefore performed a meta-analysis of the published studies to clarify this inconsistency and to establish a comprehensive picture of the relationship between *CYP2E1 *and HNSCC.

## Methods

### Identification and eligibility of relevant studies

The literature included in the current analysis was selected using PubMed with keywords "head and neck" or "oral-neoplasms" or "larynx" or "pharynx" and "cancer" or "carcinoma", "HNSCC" or "SCCHN", "cytochrome p450 2E1/IIE1", "polymorphism" or "variant" and abbreviations of the gene "Cyp2e1". No other limits were used during electronic search. All references cited in these studies and previously published review articles were reviewed to identify additional work not indexed by MEDLINE. The analyzed data is drawn from all English language publications up to August 2009.

Only those studies assessing the association between head and neck cancer and the *CYP2E1 *gene polymorphisms were included. Eligible studies had to meet the criteria: (1) They were original papers containing independent data, (2) identification of HNSCC was confirmed histologically or pathologically, (3) they provided sufficient data to calculate the odds ratio (OR) or P-value, (4) they used a case-control design, (5) they described genotyping method, equipment and protocols or made reference to them. The major reasons for exclusion of studies were (1) they were family studies, (2) they contained overlapping data, (3) they were review papers.

### Quality assessment and data extraction

Each article was read and assessed according to the score scale for randomized controlled association study proposed by Dawei et al [10]. In brief, papers were rated according to several items on the scale in relation to two areas: experiment design to minimize potential bias and data analysis. The quality score categorizes studies as of ''high'' or ''low'' quality.

For each study, the following information was extracted independently by two investigators: first author's surname, publication date, diagnosis criterion, gender, ethnicity, genotyping method, cigarette smoking status, alcohol consumption, total number of cases and controls and the numbers of cases and controls with the three genotypes. The results were compared and disagreements were discussed and resolved with consensus. Where essential information was not presented in articles, every effort was made to contact the authors.

### Statistical analysis

For the control group of each study, the distributions of genotypes were tested for Hardy-Weinberg equilibrium (HWE) using the Chi-square test. If controls of studies were found not to be in HWE, sensitivity analyses were performed with and without these studies to test the robustness of the findings. For the RsaI/PstI polymorphism, we first estimated the risks of the c1c2 and c2c2 genotypes on HNSCC, compared with the wild-type c1c1 homozygote. The risk of c1c2+c2c2 versus c1c1 on cancers was then evaluated in dominant model. The same evaluation was carried out for the DraI polymorphism. The strength of the association between the *CYP2E1 *polymorphism and head and neck cancer risk was measured by odds ratios (ORs) with 95% confidence intervals (CIs).

Cochran's χ^2 ^based Q-statistic test [11,12] and *I*^2 ^-test [13] were performed to assess possible heterogeneity in the combined studies. If heterogeneity existed, the random effects model (the DerSimonian and Laird method) [14], which yields wider confidence intervals, was adopted to calculate the overall OR value. Otherwise, the fixed effects model (the Mantel-Haenszel method) was used [15]. In addition, sources of heterogeneity were investigated by stratified meta-analyses based on ethnicity (Asian and Caucasian populations), quality (high and low) of the studies, smoking status (ever/never smokers) and alcohol consumption (users/non-users). Asian populations are mainly composed of Chinese and Japanese without Indian. 95% CIs were constructed using Woolf's method [16]. The significance of the overall OR was determined by the Z-test. Funnel plots and Egger's linear regression test were used to assess evidence for potential publication bias [17]. In order to assess the stability of the result, sensitivity analyses were performed, each study in turn was removed from the total, and the remaining were reanalyzed. The analysis was conducted using Comprehensive Meta Analysis software (Version 1.0.23, BIOSTAT, Englewood, NJ). The type I error rate was set at 0.05. All P-values were two-tailed.

## Results

### Characteristics of studies

**Figure 1:**
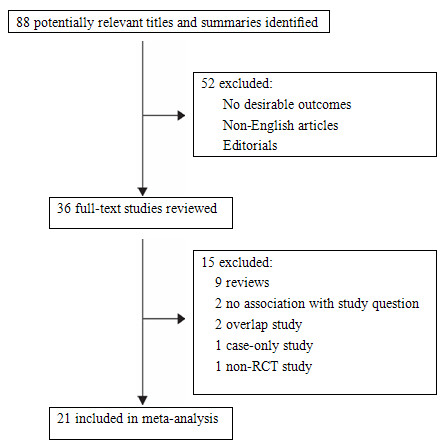
**Study selection process**.

Figure 1 shows the study selection process. The combined search yielded 88 references. After discarding overlapping references and those which clearly did not meet the criteria, 24 studies were retained. One study [18] was discarded for insufficient data (although we tried to contact the authors to query the data), two [19,20] were discarded as being studies on leukoplakia. As shown in table 1, 21 case-control studies finally met our criteria for inclusion [2,3,7,21-38]. 21 studies were identified for the *CYP2E1*-PstI/RsaI polymorphism, including a total of 4951 cases and 5995 controls, and for the DraI polymorphism 9 studies were identified covering a total of 2510 cases and 2162 controls. The detailed characteristics of these studies are summarized in table 1.

**Table 1:** Demography of the combined studies

Author, year	Polymorphism	Ethnicity	Cases	Controls	Genotype distribution in controls					
					
					PstI/RsaI			DraI		
					
					c1c1	c1c2	c2c2	TT	TA	AA
Olivieri et al.,2009 [2]	PstI/RsaI	Brazilian	153	145	105	16	0	/	/	/

Ruwali et al.,2009 [3]	PstI/RsaI, DraI	Indian	350	350	343	7	0	253	94	3

Bouchardy et al.,2000 [7]	PstI/RsaI, DraI	French Caucasians	250	172	164	8	0	151	20	1

Lucas et al.,1996 [21]	PstI/RsaI, DraI	French Caucasians	96	460	248	11	1	368	86	6

Hildesheim et al.,1997 [22]	PstI/RsaI, DraI	Taiwanese	364	320	198	113	9	183	123	14

Hung et al.,1997 [23]	PstI/RsaI	Taiwanese	41	122	76	42	4	/	/	/

González et al.,1998 [24]	PstI/RsaI	Spaish	75	200	179	21	0	/	/	/

Matthia et al.,1998 [25]	PstI/RsaI, DraI	German Caucasians	398	219	165	10	0	102	17	2

Morita et al.,1999 [26]	PstI/RsaI	Japanese	145	164	105	52	7	/	/	/

Katoh et al.,1999 [27]	PstI/RsaI	Japanese	92	146	95	45	7	/	/	/

Kongruttanachok et al.,2001 [28]	PstI/RsaI	Thai	217	297	189	103	5	/	/	/

Liu et al., 2001 [29]	PstI/RsaI	American	174	399	384	15	0	/	/	/

Neuhaus et al.,2004 [30]	PstI/RsaI, DraI	German	312	299	282	13	2	196	39	1

Li et al.,2005 [31]	PstI/RsaI	White American	724	1226	1137	86	3	/	/	/

Gajecka et al.,2005 [32]	PstI/RsaI	Poland Caucasians	289	316	305	18	0	/	/	/

Sugimura et al.,2006 [33]	PstI/RsaI, DraI	Japanese	122	241	164	70	7	126	97	18

Gattáet al.,2006 [34]	PstI/RsaI	Brazilian	103	102	96	6	0	/	/	/

Marques et al.,2006 [35]	PstI/RsaI	Brazilian	231	212	187	25	0	/	/	/

Soya et al.,2008 [36]	PstI/RsaI, DraI	Indian	408	220	212	8	0	145	65	10

Shama et al., 2008 [37]	PstI/RsaI	White American	197	416	364	39	0	/	/	/

Boccia et al.,2008 [38]	PstI/RsaI, DraI	Italian	210	245	228	15	1	224	19	1

### Meta-analysis results

### *CYP2E1*-PstI/RsaI polymorphism

Our meta-analysis gave an overall OR of 1.96 (95% CI: 1.33-2.90, P < 0.001, P_Q _= 0.87) for head and neck cancer risk among c2 homozygotes of *CYP2E1 *RsaI/PstI polymorphism compared with the wild type. However, no other genetic model gave statistically significant results.

When stratifying for ethnicity, c2 homozygotes were significantly associated with HNSCC in Asian populations with an OR of 2.08(95% CI: 1.34-3.23, P < 0.001, P_Q _= 0.52) among homozygotes (c2c2 versus c1c1). On the other hand, no such association was detected in Caucasian populations (p > 0.05 for all models). When studies were stratified for study quality, an overall OR of 1.81 (95% CI: 1.13-2.89, P = 0.013, P_Q _= 0.92) for c2 homozygotes emerged for the high-quality scored studies. These positive associations were also present among low-quality scored studies. Data on genotypes of the PstI/RsaI polymorphism among cases and controls stratified by smoking were available in five studies and by alcohol consumption in four studies. The results of the stratified meta-analyses according to smoking status and alcohol consumption are shown in Table 2. The overall ORs appeared similar in each subgroup in all three genetic models.

**Table 2:** Stratified analysis of the CYP2E1 *Rsa*I/*Pst*I polymorphism and HNSCC

Sub-grouped studies	Genetic model	No.of Studies	No.Cases/No.Controls	Test of association		Test of heterogeneity		
			
				OR (95% CI)	P	Q	P _Q_	*I*^2^
Overall	c2 carriers vs. wild	21	4892/5995	1.02(0.90-1.17)	0.68	31.37	0.05	36.26
	
	c2 homozygotes vs. wild	17	3920/5008	1.96(1.33-2.90)	<0.001	9.84	0.87	0
	
	Dominant	21	4892/5995	1.08(0.95-1.22)	0.23	30.68	0.06	34.81

Asians	c2 carriers vs. wild	6	981/1291	1.03(0.86-1.24)	0.69	5.95	0.31	16.01
	
	c2 homozygotes vs. wild	6	981/1291	2.08(1.34-3.23)	<0.001	4.17	0.52	0
	
	Dominant	6	981/1291	1.12(0.94-1.33)	0.18	4.76	0.44	0

Caucasians	c2 carriers vs. wild	15	3911/4704	1.07(0.82-1.40)	0.57	25.40	0.03	44.89
	
	c2 homozygotes vs. wild	11	2939/3717	1.58(0.68-3.68)	0.28	5.35	0.86	0
	
	Dominant	15	3911/4704	1.03(0.86-1.24)	0.49	25.49	0.03	45.09

High quality	c2 carriers vs. wild	16	3914/4688	1.08(0.86-1.35)	0.48	26.49	0.033	43.38
	
	c2 homozygotes vs. wild	13	3173/3913	1.81(1.13-2.89)	0.013	5.76	0.92	0
	
	Dominant	16	3914/4688	1.11(0.90-1.37)	0.29	24.09	0.064	37.74

Low quality	c2 carriers vs. wild	5	978/1307	1.07(0.84-1.36)	0.57	4.71	0.31	15.11
	
	c2 homozygotes vs. wild	4	747/1095	2.36(1.16-4.79)	0.017	3.70	0.29	19.04
	
	Dominant	5	978/1307	1.12(0.81-1.55)	0.46	6.38	0.17	37.39

Ever-smokers	c2 carriers vs. wild	5	1173/831	0.62(0.34-1.12)	0.11	8.67	0.07	53.88
	
	c2 homozygotes vs. wild	2	385/325	0.47(0.15-1.47)	0.19	0.10	0.74	0
	
	Dominant	5	1173/831	0.61(0.33-1.11)	0.10	8.93	0.063	55.19

Never-smokers	c2 carriers vs. wild	4	549/677	1.40(0.93-2.11)	0.09	0.44	0.93	0
	
	c2 homozygotes vs. wild	3	441/431	3.25(0.48-21.70)	0.22	3.66	0.16	45.43
	
	Dominant	4	549/677	1.45(0.98-2.16)	0.06	1.19	0.75	0

Alcohol- drinkers	c2 carriers vs. wild	4	565/467	1.12(0.51-2.43)	0.76	8.39	0.039	64.26
	
	c2 homozygotes vs. wild	2	198/296	1.48(0.34-6.44)	0.59	0.83	0.36	0
	
	Dominant	4	565/467	1.11(0.53-2.29)	0.77	7.58	0.055	60.43

Non-drinkers	c2 carriers vs. wild	3	653/883	0.92(0.64-1.33)	0.69	1.31	0.51	0
	
	c2 homozygotes vs. wild	2	443/370	2.22(0.93-5.30)	0.07	0.004	0.94	0
	
	Dominant	3	653/883	1.07(0.76-1.52)	0.67	1.08	0.58	0

### *CYP2E1*-DraI polymorphism

We also carried out a comprehensive meta-analysis of *CYP2E1 *DraI polymorphisms for each ethnic group and for the different quality grades of studies under various genetic models (Table 3). The overall OR of head and neck cancer was 1.56(95% CI: 1.06-2.27, P = 0.021, P_Q _= 0.37) for homozygotes compared with wild genotype, but no association was observed between T7632A and head and neck cancer using other genetic models.

**Table 3:** Stratified analysis of the CYP2E1 *Dra*I polymorphism and HNSCC

Sub-grouped studies	Genetic model	No.of Studies	No.Cases/No.Controls	Test of association		Test of heterogeneity		
				
				OR (95% CI)	P	Q	P _Q_	*I*^2^
Overall	AT vs. TT	9	2407/2126	1.15(0.90-1.47)	0.25	19.92	0.011	59.84
	
	AA vs. TT	9	2407/2126	1.56(1.06-2.27)	0.021	8.62	0.37	7.21
	
	Dominant	9	2407/2126	1.18(0.94-1.49)	0.13	18.81	0.016	57.47

Asians	AT vs. TT	2	486/561	0.92(0.70-1.19)	0.53	0.13	0.71	0
	
	AA vs. TT	2	486/561	1.99(1.22-3.24)	0.006	0.067	0.79	0
	
	Dominant	2	486/561	1.04(0.81-1.34)	0.72	0.37	0.53	0

Caucasians	AT vs. TT	7	1921/1601	1.24(0.92-1.66)	0.15	14.61	0.02	58.95
	
	AA vs. TT	7	1921/1601	1.08(0.59-1.97)	0.78	6.18	0.40	2.98
	
	Dominant	7	1921/1601	1.22(0.90-1.65)	0.18	16.29	0.01	63.17

High quality	AT vs. TT	7	2189/1663	1.12(0.83-1.51)	0.43	18.33	0.005	67.27
	
	AA vs. TT	7	2189/1663	1.28(0.81-2.03)	0.28	5.89	0.43	0
	
	Dominant	7	2189/1663	1.13(0.85-1.49)	0.38	17.11	0.009	64.93

Low quality	AT vs. TT	2	218/499	1.19(0.82-1.72)	0.35	1.59	0.20	37.10
	
	AA vs. TT	2	218/499	2.36(1.20-4.63)	0.012	0.56	0.45	0
	
	Dominant	2	218/499	1.35(0.95-1.92)	0.085	1.25	0.26	20.00

After stratification for ethnicity, we observed an OR of 1.99(95% CI: 1.22-3.24, P = 0.006, P _Q _= 0.79) for the DraI polymorphism in Asian populations among homozygotes (AA versus TT), whereas no such association was observed in Caucasian populations. After stratification for study quality, an overall OR of 2.36 (95% CI: 1.20-4.63, P = 0.012, P_Q _= 0.45) for homozygotes emerged for the low-quality scored studies but no such association was observed among high-quality studies (Table 3).

### Sensitivity analyses and Publication bias

Most studies indicated that the frequency distributions of genotypes in the controls were consistent with Hardy-Weinberg equilibrium (HWE), whereas deviations from HWE were observed in three studies of the PstI/RsaI polymorphism [21,28,30]. However, the corresponding pooled ORs were not substantially altered whether or not these studies were included (Data not shown). In addition, sensitivity analysis indicated that no single study influenced the pooled OR qualitatively, suggesting that the results of this meta-analysis are stable.

Begger's funnel plot and Egger's test were used to assess publication bias. The shape of the funnel plots was symmetrical (Additional file 1). The Egger test provided evidence that there was no publication bias among the studies included (p > 0.05, for all).

## Discussion

In the present meta-analysis, we examined the association between two widely studied *CYP2E1 *polymorphisms (PstI/RsaI, DraI) and head and neck cancer risk. We found that the variant homozygote of the *CYP2E1 *PstI/RsaI polymorphism was significantly associated with cancer risk in the overall comparisons, compared with the wild homozygote. We also observed a significant association between the *CYP2E1 *DraI polymorphism and cancer risk for homozygote in the overall comparisons.

As for the PstI/RsaI polymorphism of *CYP2E1*, our result showed a significantly high cancer risk for the c2 homozygote in Asian populations, with no such association being found among Caucasian population under any of the three genetic models. Ethnic differences may attribute to these different results, since the distributions of the less common c2 allele were different between various races, with a prevalence of ~25-50% and 5-10% among Asians and Caucasians, respectively [39]. The c2 variant allele frequency did reach a statistically significant level among Asians, while in the Caucasian populations, the lack of significant association might be explained by the substantially lower statistical power caused by the lower prevalence of *CYP2E1 *c2 allele (5-10% against 25-50% for Asians).

*CYP2E1 *is presumed to confer susceptibility to HNSCC by metabolizing carcinogens but, unfortunately, few of the studies explored the interaction between the *CYP2E1 *genotype and smoking habits or alcohol consumption. This may be due to the low statistical power of the individual studies to detect such interactions; but all the studies which collected these data were utilized for this meta-analysis. The stratified meta-analyses of all the data on the PstI/RsaI genotype and smoking habits or alcohol consumption with respect to head and neck cancer risk produced no statistically significant results. These results suggest that when the environmental and genetic risk factors are both present, the combined effect on head and neck cancer seems to be no longer than additive of the separate effects. However, we cannot ignore the fact that owing to the low prevalence of c2 homozygotes in each study, even when data are pooled, the statistical power to detect an interaction remains low.

In addition, the DraI polymorphism was associated with an increased cancer risk among Asians but not among Caucasians, suggesting an ethnic difference in terms of genetic and environmental factors [40]. However, only two studies of the DraI polymorphisms used Asian population data and it is therefore probable that the observed ethnic differences were simply due to chance, given that studies with small sample size may have insufficient statistical power to detect a slight effect. Additional studies are therefore required to further validate ethnic differences in the effect of DraI polymorphisms on cancer risk, especially in Asian populations.

Currently, the mechanism whereby the rare allele of the RsaI/PstI and DraI polymorphisms increases the risk of head and neck cancer risk are still not clear. *CYP2E1 *is a phase I enzyme, which plays an essential role in the metabolic activation of low molecular weight compounds and pro-carcinogens such as N-nitrosamines, benzene, and halogenated hydrocarbons. There is strong evidence from in vitro studies suggesting that the rare allele of the RsaI/PstI polymorphisms in the *CYP2E1 *gene probably confers a higher risk of HNSCC susceptibility by increasing transcriptional and enzyme activity [6]. Thus, the c2/c2 genotypes may be more liable to metabolically activate mutagens and carcinogens. As for the rare allele of the DraI polymorphism of the *CYP2E1 *gene, in vitro expression studies indicate that it is associated with increased transcriptional activity [9]. The AA genotypes may therefore have more ability to metabolize mutagens and pro-carcinogens.

Some limitations should be considered when interpreting our results, in addition to those inherited from the meta-analysis. First, our results are based on unadjusted estimates, whereas adjustments for factors such as age and sex would produce a more precise analysis. The lack of this kind of information may cause serious confounding bias. Further large and well-designed studies need to be performed to further confirm all these results. Secondly, the subgroup meta-analyses dealing with interactions between the *CYP2E1 *genotype and smoking habits/alcohol consumption are based on the small number of studies where such information is available. Nevertheless the number of subjects included in this part of the analysis comprised the largest sample of all. Third, the quality score of the individual studies included in our meta-analysis was assessed on the basis of trying to minimize the potential for selection bias, misclassification related to exposure, collection of data on potential confounders and method of statistical analysis. No validated quality assessment system currently exists, and it is evident that our quality scale has a subjective component.

## Conclusions

In summary, this may be the first systematic and comprehensive meta-analysis of *CYP2E1 *and HNSCC. Our results indicate that the *CYP2E1*-PstI/RsaI and DraI polymorphisms may be a risk factor for head and neck cancer in Asian populations, and particularly for individual homozygotes for the unfavourable gene variant. However additional large studies are needed to validate our findings. Future studies should use standardized unbiased genotyping methods as well as homogeneous cancer patients and well-matched controls.

## Abbreviations

HNSCC: squamous cell carcinoma of head and neck; OR: odds ratio; CI: confidence interval; HWE: Hardy-Weinberg equilibrium.

## Competing interests

The authors declare that they have no competing interests.

## Authors' contributions

KFT organized all the research, performed data analysis and drafted the manuscript. YL collected the articles, co-worked in data analysis, as well as manuscript preparation. ZZ participated in the calculation and table generation, and helped to draft the manuscript. YMG and YYX participated in the data extraction and manuscript editing. GYF, LH and SYQ provided advice for preparing the manuscript. All authors read and approved the final manuscript.

## Pre-publication history

The pre-publication history for this paper can be accessed here:

http://www.biomedcentral.com/1471-2407/10/575/prepub

## Supplementary Material

Additional file 1 **T-test and Funnel plot**. Egger's test and Begger's funnel plot results to assess publication bias. Click here for file
