# Supplementary material for: The PstI/RsaI and DraI polymorphisms of CYP2E1 and head and neck cancer risk: a meta-analysis based on 21 case-control studies
Source: BMC Cancer. 2010 Oct 22;10:575. doi: 10.1186/1471-2407-10-575 (PMC2988025; doi:10.1186/1471-2407-10-575)
Supplement: Additional file 1 — T-test and Funnel plot. Egger's test and Begger's funnel plot results to assess publication bias. [file 1471-2407-10-575-S1.DOC]

**T test**

RsaI/PstI Polymorphism

c1c2 vs. c1c1 *P*(T)=0.087

c2c2 vs. c1c1 *P*(T)=0.336

Dominant *P*(T)=0.202

DraI Polymorphism

AT vs. TT *P*(T)=0.469

AA vs. TT *P*(T)=0.373

Dominant *P*(T)=0.467

*P*(T): T test used to evaluate the significance of publication bias.

**Funnel plot**

**PstI/RsaI polymorphism**

Begg’s funnel plot of *CYP2E1* RsaI/PstI polymorphism and head and ncek cancer risk for the c2 heterozygote versus c1/c1.

Begg’s funnel plot of *CYP2E1* RsaI/PstI polymorphism and head and neck cancer risk for the c2 homozygote versus c1/c1.

Begg’s funnel plot of *CYP2E1* RsaI/PstI polymorphism and head and neck cancer risk using dominant model (c1c2 + c2c2 versus c1c1).

**DraI polymorphism**

Begg’s funnel plot of *CYP2E1* DraI polymorphism and head and neck cancer risk for the c2 heterozygote versus c1/c1.

Begg’s funnel plot of *CYP2E1* DraI polymorphism and head and neck cancer risk for the c2 homozygote versus c1/c1.

Begg’s funnel plot of *CYP2E1* DraI polymorphism and head and neck cancer risk using dominant model (c1c2 + c2c2 versus c1c1).
